# Presence of cerebrospinal fluid antibodies associated with autoimmune encephalitis of humans in dogs with neurologic disease

**DOI:** 10.1111/jvim.15616

**Published:** 2019-09-08

**Authors:** Emma G. Stafford, Amanda Kortum, Aude Castel, Lauren Green, Jeanie Lau, Peter J. Early, Karen R. Muñana, Christopher L. Mariani, Jeffrey A. Yoder, Natasha J. Olby

**Affiliations:** ^1^ Department of Population Health and Pathobiology, College of Veterinary Medicine North Carolina State University Raleigh North Carolina; ^2^ Comparative Medicine Institute North Carolina State University Raleigh North Carolina; ^3^ Clinical Veterinary Pharmacy, College of Veterinary Medicine North Carolina State University Raleigh North Carolina; ^4^ Department of Molecular Biomedical Sciences, College of Veterinary Medicine North Carolina State University Raleigh North Carolina; ^5^ Department of Small Animal Clinical Sciences, University of Tennessee Veterinary Teaching Hospital College of Veterinary Medicine Knoxville Tennessee; ^6^ Department of Clinical Sciences, College of Veterinary Medicine North Carolina State University Raleigh North Carolina

**Keywords:** immunofluorescent assay, meningoencephalitis, MUE, NMDA receptor encephalitis

## Abstract

**Background:**

Presumed autoimmune diseases affecting the central nervous system (CNS) of dogs are common. In people, antibodies against neuronal cell surface antigens that are associated with a wide variety of neurological syndromes have been identified. The presence of cerebrospinal fluid (CSF) autoantibodies that target neuronal cell surface proteins has not been reported in dogs with neurologic disorders.

**Objectives:**

Autoantibodies to neuronal cell surface antigens can be found in the CSF of dogs with inflammatory CNS disease. Our aim was to determine whether 6 neuronal cell surface autoantibodies were present in the CSF of dogs diagnosed with inflammatory and noninflammatory CNS disease.

**Animals:**

Client‐owned dogs with CNS disease and complete diagnostic evaluation including magnetic resonance imaging and CSF analysis were included. One healthy dog was included as a negative control.

**Methods:**

Cerebrospinal fluid was tested for 6 antigenic targets with a commercially available indirect immunofluorescence assay test kit.

**Results:**

There were 32 dogs with neurological disease, 19 diagnosed with inflammatory disease (encephalitis and meningitis), 10 with noninflammatory disease (neoplasia, intervertebral disk disease, degenerative myelopathy, and epilepsy), 2 with no diagnosis, and 1 with neoplasia and meningoencephalitis. Anti‐N‐methyl‐d‐aspartate receptor 1 (NMDAR1) antibodies were detected in 3 dogs (3/32; 9.38%). All 3 dogs responded to treatment of meningoencephalomyelitis of unknown etiology (MUE).

**Conclusions and Clinical Importance:**

Further evaluation of the prevalence and clinical relevance of CSF and serum antibodies to neuronal cell surface antigens is warranted. Defining antigenic targets associated with encephalitis in dogs might allow diagnostic categorization of MUE antemortem.

**Abbreviations**
AE: autoimmune encephalitis
AMPA: α‐amino‐3‐hydroxy‐5‐methyl‐4‐isoxazoleproprionic acid
CASPR: contactin‐associated protein
CNS: central nervous system
CSF: cerebrospinal fluid
DPPX: dipeptidyl aminopeptidase‐like protein
GABA_B_: gamma‐aminobutyric acid type B
GME: granulomatous meningoencephalomyelitis
IF: immunofluorescence
IFA: immunofluorescent assay
LGI1: leucine‐rich glioma‐inactivated protein 1
MRI: magnetic resonance imaging
MUE: meningoencephalomyelitis of unknown etiology
NMDA: N‐methyl‐D‐aspartate
NMDAR: N‐methyl‐D‐aspartate receptor
NMDARE: NMDA receptor encephalitis
PBS: phosphate‐buffered saline
PBST: phosphate‐buffered saline containing 0.2% Tween‐20
VGKC: voltage‐gated potassium channel
VGCC: voltage‐gated calcium channel
PCR: polymerase chain reaction

## 1 INTRODUCTION

Meningoencephalomyelitis is a common cause of neurological disease in dogs, with small breed, young adult dogs particularly at risk.^1^, ^2^ Although some cases are caused by central nervous system (CNS) infections, most cases do not have an identifiable underlying infection and are considered immune‐mediated.^2^, ^3^ Several different syndromes are recognized, including a primarily cerebellar syndrome (idiopathic cerebellitis or generalized tremor syndrome),^4^, ^5^, ^6^ eosinophilic meningoencephalitis,^7^, ^8^, ^9^ and encephalitides defined as necrotizing or granulomatous histopathologically.^2^ Because of difficulty in defining these latter conditions antemortem, the term “meningoencephalomyelitis of unknown etiology” (MUE) has been used. This umbrella term includes meningoencephalomyelitis diagnosed by a combination of magnetic resonance imaging (MRI) and cerebrospinal fluid (CSF) findings and negative infectious disease testing.^1^, ^2^ Extensive efforts to identify an underlying infectious cause for these idiopathic inflammatory conditions have failed, and they are managed by immunosuppression.^3^, ^10^

In people, the description of antibodies to neuronal cell surface or synaptic proteins has revolutionized the understanding of neurologic disorders in recent years.^11^ Syndromes previously defined as “idiopathic” have been characterized as autoimmune by the identification of antibodies to a variety of self‐proteins.^12^, ^13^ The most famous example, due in part to a book and the film it inspired, entitled “Brain on Fire,” is N‐methyl‐d‐aspartate receptor encephalitis (NMDARE).^14^ This disease can present with a range of neurologic signs including dyskinesia and seizures and it is now recognized as a characteristic clinical syndrome having an association, in 40% of patients, with teratoma.^13^, ^15^, ^16^ However, psychiatric signs can dominate, particularly in younger patients, resulting in misdiagnosis and mismanagement.^13^ It can be diagnosed now by detection of antibodies in the CSF and serum, and patients with NMDARE who receive early and aggressive immunotherapy have an excellent prognosis with >80% of patients returning to their baseline functioning.^15^, ^16^ The prevalence of these autoimmune disease diagnoses in people has increased dramatically because commercial antibody testing has become available, with NMDARE becoming the most common noninfectious encephalitis in people.^17^, ^18^

In veterinary medicine, antibodies against glial fibrillary acidic protein (GFAP), an intracellular astrocytic protein, have been identified in the CSF of dogs with granulomatous meningoencephalomyelitis (GME) and necrotizing meningoencephalitis.^19^, ^20^ In addition, anti‐NMDAR autoantibodies were identified in the CSF of a captive polar bear after it died following a generalized seizure,^21^ and increased serum concentrations of antivoltage‐gated potassium channel (VGKC) complex, specifically anti‐leucine‐rich glioma‐inactivated protein 1 (anti‐LGI1) antibodies have been identified in cats with limbic epilepsy,^22^ raising the question of whether antigenic targets identified in encephalitis of humans could be associated with encephalitis in other mammals. We hypothesized that antibodies to neuronal surface proteins would be present in the CSF of dogs with inflammatory, noninfectious CNS disease. Our objective was to determine the prevalence of 6 autoantibodies associated with autoimmune encephalitis (AE) of humans in CSF of dogs with inflammatory and noninflammatory CNS disease.

## 2 METHODS

### 2.1 Bioinformatics: Protein sequence comparison

A BLASTp analysis using the following human proteins as a query in the National Center for Biotechnology Information database was performed to identify canine homologs: glutamate receptor ionotropic NMDA1 receptor (NMDAR1/GRIN1), alpha‐amino‐3‐hydroxy‐5‐methyl‐4‐isoxazoleproprionic acid receptors 1 and 2 (AMPAR1/R2), gamma‐aminobutyric acid type B receptors 1 and 2 (GABA_B_R1/R2), LGI1, contactin‐associated protein receptor 2 (CASPR2), and dipeptidyl aminopeptidase‐like protein (DPPX)^23^; see Table 1 for accession numbers. Human and canine NMDAR1 sequences were aligned using T‐Coffee.^24^

**Table 1:** Sequence homology between dog and human protein targets of each antibody

Human protein	Symbol	GenBank ID	Top Canine BLASTp hit (*Canis lupus familiaris*)	Top BLASTp protein ID	Query cover (BLASTp) (%)	Homology (BLASTp) (%)
Glutamate receptor ionotropic, NMDA 1 isoform GluN1‐1a precursor	NMDAR1/GRIN1	NP_015566.1	Glutamate receptor ionotropic, NMDA 1 precursor	NP_001008717	95	99
Gamma‐aminobutyric acid type B receptor subunit 1 isoform a precursor	GABA_B_R1	NP_001461.1	Gamma‐aminobutyric acid type B receptor subunit 1	XP_022270239	92	98
Gamma‐aminobutyric acid type B receptor subunit 2 precursor	GABA_B_R2	NP_005449.5	Gamma‐aminobutyric acid type B receptor subunit 2	XP_538749.2	100	99
Glutamate receptor 1 isoform 1 precursor	AMPAR1	NP_000818.2	Glutamate receptor 1 isoform X2	XP_853398.1	100	99
Glutamate receptor 2 isoform 1 precursor	AMPAR2	NP_000817.2	Glutamate receptor 2 isoform X1	XP_013975091.1	92	99
Dipeptidyl aminopeptidase‐like protein 6 isoform 3	DPPX	NP_001034439.1	Dipeptidyl aminopeptidase‐like protein 6 isoform X4	XP_005629758.1	98	94
Leucine‐rich glioma‐inactivated protein 1 isoform 2 precursor	LG1	NP_001295204.1	Leucine‐rich glioma‐inactivated protein 1 isoform X3	XP_013964506.1	100	99
Contactin‐associated protein‐like 2 precursor	CASPR2	NP_054860.1	Contactin‐associated protein‐like 2 isoform X1	XP_003432128.1	100	95

### 2.2 Cerebrospinal fluid samples

Cerebrospinal fluid samples were selected from the Neurology Service CSF bank at North Carolina State University College of Veterinary Medicine. These samples were obtained from dogs undergoing diagnostic evaluation for CNS disorders. Samples were obtained from the cerebellomedullary or lumbar cistern and submitted for routine analysis (measurement of protein concentration, red and white blood cell counts, and cytology). Residual CSF then was stored at −80°C within 24 hours of sampling according to the immunofluorescence assay (IFA) manufacturer's directions. Samples were selected from dogs with neurological signs localizing to the brain or spinal cord that also had brain or spinal cord MRI and CSF analysis. Clinical details of all dogs were extracted from their medical records and the final diagnosis and treatment were recorded. All dogs diagnosed with MUE also had negative infectious disease testing, which included negative polymerase chain reaction (PCR) of blood and CSF for canine distemper virus, West Nile virus, *Borrelia burgdorferi*, *Neospora hughesi*, *Neospora caninum*, *Toxoplasma gondii*, *Anaplasma phagocytophilum*, *Ehrlichia canis*, and *Rickettsia* spp. (Canine Neurological Panel, Real‐time PCR Research and Diagnostics Core Facility, University of California‐Davis, California). Additional serology was performed in certain cases at the discretion of the clinician. Cerebrospinal fluid banked from a healthy, neurologically normal beagle dog taking part in a separate research study served as the negative control. All research samples were acquired in accordance with guidelines and approval of the North Carolina State University Institutional Animal Care and Use Committee (protocol number: 17‐053‐O).

### 2.3 Immunofluorescence assay

Autoimmune Encephalitis Mosaic 6 testing kit (EUROIMMUN, Lübeck, Germany, FA 112d‐1003‐6) is an indirect IFA used to screen for autoantibodies against 6 targets associated with autoimmune encephalitic diseases of humans.^25^ It tests for antibodies against NMDAR1, AMPAR1, AMPAR2, GABA_B_R1, and GABA_B_R2 receptors, along with other protein targets: CASPR2, LGI1, and DPPX. To minimize background signal in samples from dogs with severe inflammatory conditions, CSF samples with protein concentration >50 mg/dL were diluted to a final protein concentration of 50 mg/dL with ×1 phosphate‐buffered saline (PBS) containing 0.2% Tween‐20 (PBST). Cerebrospinal fluid samples were applied to the kit's proprietary “biochip” according to the manufacturer's instructions. An anti‐human NMDAR1 antibody, supplied by the manufacturer, was used as a positive control. A sample from a healthy beagle dog was included in each technical replicate as a negative control. After incubation, samples were washed with ×1 PBST and secondary antibodies were applied according to manufacturer's instructions. Secondary antibodies provided by the manufacturer included a fluorescein‐labeled goat anti‐dog IgG (EUROIMMUN, Lübeck, Germany, AF 102‐0115 C) applied to all canine samples and a fluorescein‐labeled goat anti‐human antibody (EUROIMMUN, FA 112d‐1003‐6) was used for the positive control. Biochips were incubated and washed with PBST. Fluorescent and differential interference contrast (DIC) images were acquired with a Leica DM5000B fluorescent microscope at ×20. Each reaction field was captured in 4 quadrants using the same imaging technique. Images were reviewed for the presence of immunofluorescent cells. Manufacturer's instructions note that not all cells are transfected with the antigen, thus the presence of immunofluorescence (IF) is not expected of all cells within a field. The characteristics of cellular IF differ among the antigens and a full description of expected staining patterns was provided by the manufacturer and used to determine the presence of positive staining. Samples from dogs that had positive results were retested, with positive and negative controls, to ensure the results were repeatable.

## 3 RESULTS

### 3.1 Conservation of protein sequences

**Figure 1:**
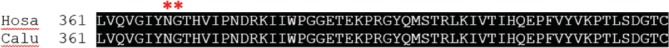
Amino acid sequence for NMDAR1 in people (*Homo sapiens*; Hosa) and dogs (*Canis lupus familiaris*; Calu) starting at amino acid 361. Critical amino acid positions 368 and 369. Asterisks (*) represent conservation of amino acids responsible for immunogenicity in human NMDARE. NMDAR1, N‐methyl‐d‐aspartate receptor 1; NMDARE, NMDAR encephalitis

The NMDA receptor is made of 3 protein subunits, NMDAR1, NMDAR2, and NMDAR3.^26^ The epitope of NMDAR autoantibodies in people is located on the NMDAR1 subunit, specifically at amino acids 368B and 369G.^16^, ^27^ A canine NMDAR1 homolog was identified as being 99% similar to the longest human NMDAR1 isoform (Table 1). Alignment of the canine and human NMDAR1 protein sequences indicated that the critical 368N and 369G immunogenic epitopes were conserved in dogs (Figure 1). For AMPAR1/R2, GABA_B_R1/R2, LGI1, CASPR2, and DPPX, high similarities (94‐99%) were observed between the human and putative canine homologs (Table 1).

### 3.2 Animals

Thirty‐two dogs with CNS disease and 1 healthy beagle dog were included in the study. Full details of each dog's presenting signs, diagnostic findings, and treatment are provided in Table S1. The study included 4 Maltese, 4 Golden Retrievers, 3 Poodles (Toy/Standard/Miniature), 3 mixed breeds, 2 French Bulldogs, 2 Dachshunds, 2 Labrador Retrievers, and 1 each of the following breeds or breed mixes: Beagle, Cavalier King Charles Spaniel, Peekapoo, Yorkiepoo, Goldendoodle, Labradoodle, Boston Terrier, Bernese Mountain Dog, Scottish Terrier, Japanese Chin, American Staffordshire Terrier, and Schnauzer. Of these dogs, 13 were female (all spayed) and 19 were male (all neutered) and they ranged from 8 months to 11 years in age with a mean age of 6.2 years (SD, 3.1). Nineteen dogs were diagnosed with noninfectious inflammatory disease of the CNS: 1 with idiopathic cerebellitis, 1 with steroid‐responsive meningitis arteritis (SRMA), and 17 with an antemortem diagnosis or suspicion of MUE. Three of these 17 dogs underwent necropsy with histopathological diagnoses of GME (n = 3). One additional dog, a 1‐year‐old spayed female French bulldog, presented with multifocal CNS signs, but neurodiagnostic test results were normal. This dog responded to 1 mg/kg prednisone daily as well as continued treatment of previously diagnosed tooth root abscesses with antibiotics. Ten dogs were diagnosed with noninflammatory CNS conditions. Three of these dogs were diagnosed with idiopathic epilepsy, 1 with unknown epilepsy, 4 with suspected or confirmed brain neoplasia (1 gliomatosis cerebri, 1 anaplastic oligodendroglioma, and 2 presumptive meningiomas based on imaging characteristics), 2 with thoracolumbar intervertebral disk extrusions, and 1 with degenerative myelopathy. One dog had a brain mass most consistent with meningioma but had profound neutrophilic pleocytosis and was diagnosed with both suspected neoplasia and neutrophilic pleocytosis. The final dog had a differential diagnosis of MUE, round cell neoplasia, or gliomatosis cerebri based on MRI appearance and CSF analysis, but was euthanized with no necropsy, and a final diagnosis was not established.

### 3.3 Immunofluorescent assay results

**Figure 2:**
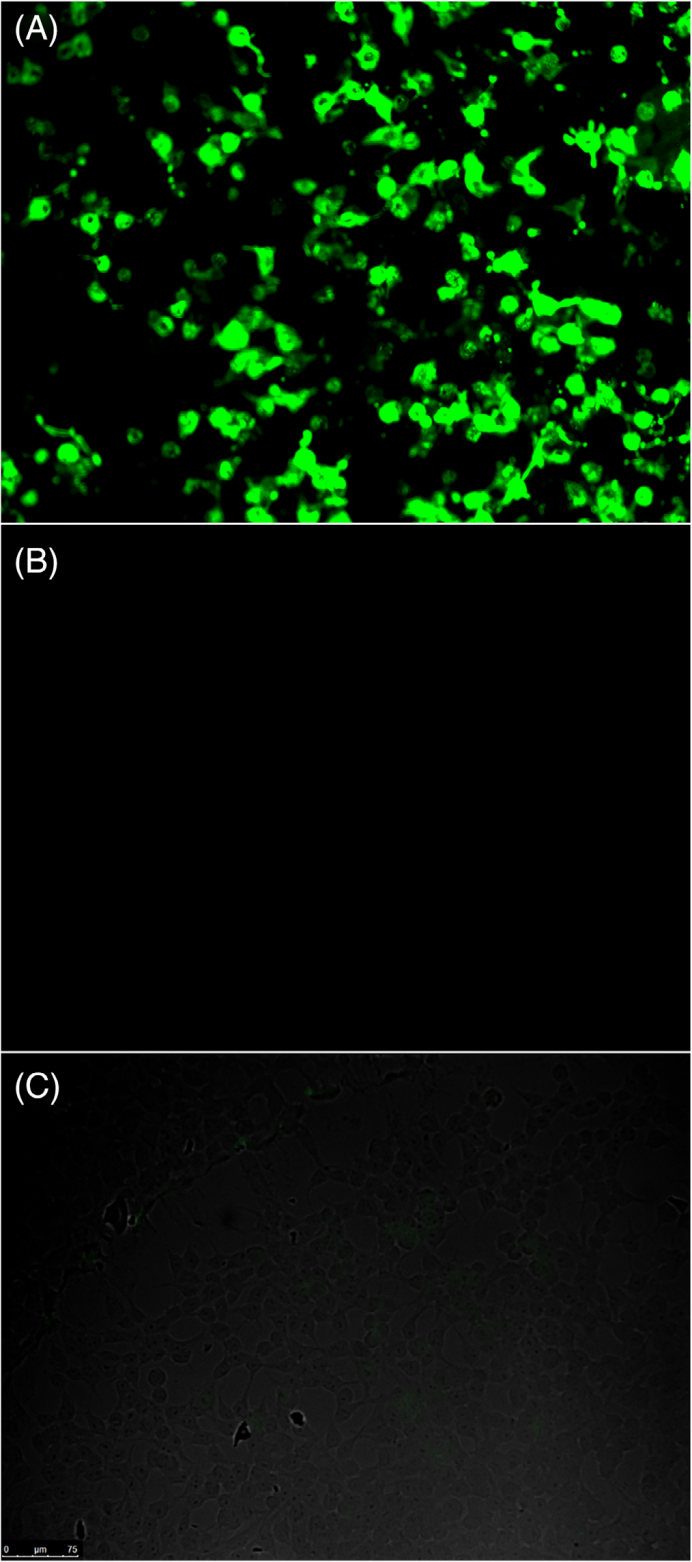
Immunofluorescent assay results for the presence of CSF anti‐NMDAR1 antibodies. A. Human positive control. B. Negative results in a healthy dog. C. Negative results in dog 3. CSF, cerebrospinal fluid; NMDAR1, N‐methyl‐d‐aspartate receptor 1

**Figure 3:**
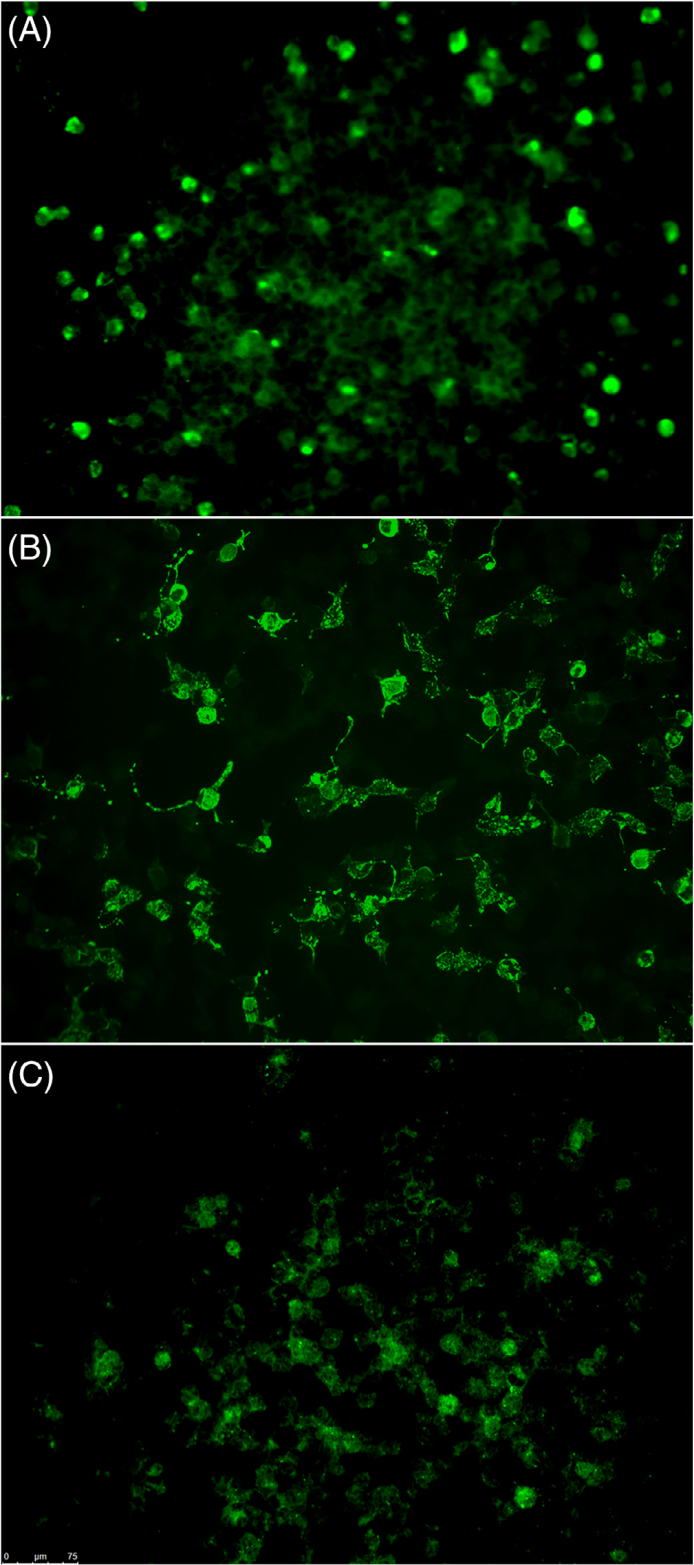
Immunofluorescent assay results for the presence of CSF anti‐NMDAR1 antibodies in 3 dogs with positive results. A. Dog 4. B. Dog 17. C. Dog 10. The intensity of staining is lower in the positive dog samples than the human control sample but there is clear positive staining in each sample. NMDAR1, N‐methyl‐d‐aspartate receptor 1

The IFA results demonstrated a high level of IF for the positive NMDAR1 control as expected, whereas a lack of IF was observed for all antibodies using the healthy canine sample (Figure 2). Of the 6 antibodies, the only positive IF was seen to NMDAR1 in 3 dogs (Figure 3), whereas the 5 other antibodies were negative in all dogs. The 3 positive NMDAR1 dogs included a 6‐year‐old spayed female miniature poodle diagnosed with MUE, a 1‐year‐old castrated male standard poodle diagnosed with MUE, and a 1‐year‐old spayed female French bulldog diagnosed with a tooth root abscess (dogs 4, 10, and 17, respectively, in Table S1). All 3 dogs presented with multifocal CNS signs. Dog 4 had both CSF pleocytosis and inflammatory lesions on brain MRI (Table S1) with negative infectious disease testing, and responded well to immunosuppression with prednisone using a 2mg/kg/day PO tapering course (Westward Pharmaceuticals, Eatontown, NJ) and cytosine arabinoside 300 mg/m^2^ SC every 3 weeks for 8 doses (Zydus, Hopewell Township, NJ; Fresenius Kabi, Bad Homburg, Germany). At the time of writing, the dog was alive and neurologically normal 22 months after diagnosis and was being treated with 0.33 mg/kg of prednisone PO q24h. Decrease in prednisone below this dose resulted in neurologic deterioration. Dog 10 had normal MRI results but CSF pleocytosis (Table S1) with negative infectious disease testing and responded well to immunosuppression with a 24‐week tapering course of prednisone starting at 2 mg/kg/day (HIKMA Pharmaceuticals, Amman, Jordan). The dog was normal at time of writing and not on any treatment.

Dog 17 presented with a signalment and signs of multifocal CNS dysfunction typical of dogs with MUE, but with a complex history. Nine days before presentation at our institution, the dog presented to the primary veterinarian with a 3‐day history of coughing and snorting. Thoracic radiographs and urinalysis were performed and were normal. The dog was treated with marbofloxacin 4 mg/kg PO q24h and carprofen 2.6 mg/kg PO q24h. The next day, the dog's breathing appeared to deteriorate, and it started to circle, wander, and bark at random objects as well as stumble unexpectedly. The dog was anesthetized by the family veterinarian who performed brachycephalic airway syndrome surgery. The veterinarian also extracted 3 abscessed maxillary teeth and noted purulent discharge. Treatment with marbofloxacin and carprofen was continued and metronidazole was added for the next 2 days for diarrhea that developed after surgery. During this time, the dog developed circling to the right and was referred to North Carolina State University Veterinary Teaching Hospital. On presentation, the dog had serosanguinous discharge from the left nostril, ptyalism, and frequent snorting with normal lung sounds and eupnea. On neurological examination, the dog was dull and intermittently disoriented with a gait characterized by wide circles to the right and a right head tilt with postural reaction deficits in the right thoracic and pelvic limbs. The dog's cranial nerve examination was unremarkable, with the exception of the head tilt. The clinical signs were localized to the right forebrain and brainstem. No abnormalities were identified in the brain on MRI, but the left maxilla was abscessed with involvement of the left nasal cavity. There was no evidence of extension of this pathology into the cranial cavity. Cerebrospinal fluid from the cerebellomedullary cistern was within normal limits. Infectious disease testing was negative, and a bile acid tolerance test was within normal limits. Given the presenting signs, breed, and age, the dog was treated with a 6‐week tapering course of prednisone (Westward Pharmaceuticals) starting at 1 mg/kg/day while also treating the tooth root abscesses with clindamycin 15.6 mg/kg PO q12h for 21 days (Ohm Laboratories, New Brunswick, NJ) and minocycline 10 mg/kg PO q12h for 21 days (Aurobino, Dayton, New Jersey). Clinical signs initially resolved but recurred as the prednisone dose was tapered. The dose was increased back to 1 mg/kg/day and then tapered again over a 3‐month period and signs resolved.

## 4 DISCUSSION

We screened CSF from 32 dogs diagnosed with a variety of neurologic disorders with a commercially available IFA kit that detects antibodies to 6 different neuronal cell surface or synaptic targets associated with AE in humans. Cerebrospinal fluid samples from 3 dogs were positive by IF indicating that antibodies against NMDAR were present, whereas all other antibody targets were negative in all dogs. Two of the 3 dogs that were positive for anti‐NMDAR antibodies were diagnosed with MUE and responded to immunosuppression. A specific diagnosis was not established in the third dog, but this dog responded to a combination of antibiotics and prednisone treatment. Our results suggest that autoantibodies against NMDAR deserve further investigation for their role in neurologic signs in dogs with encephalitis.

Autoimmune encephalitis is an increasingly important diagnosis in people since the first compelling description of the presence of antibodies against CNS neuronal surface antigens in 6 patients with limbic epilepsy in 2005.^28^ Antibodies that target neuronal cell surface proteins are considered distinct from antibodies against intracellular neuronal antigens, such as anti‐Hu antibodies. Antibodies against intracellular neuronal antigens have been recognized for much longer, are always associated with cancer, and typically are associated with neurologic dysfunction resulting from irreversible T‐cell cytotoxicity.^11^ Since 2005, >20 different neuronal cell surface antigenic targets have been identified and associated with a number of neurologic conditions.^11^ These include such diverse entities as limbic epilepsy, neuropsychiatric disorders, memory loss, cerebellar ataxia, chronic pain, and nystagmus.^29^ The most common antigenic targets include NMDAR, a complex of 2 proteins closely associated with the VGKC, namely, LGI1 and CASPR2, voltage ‐gated calcium channels (VGCC), the metabotropic glutamate receptors 1 and 5 (mGluR1, mGluR5) as well as AMPA and GABA_B_ (see Table S2 for details). Although cancer is not always present, several of these autoantibodies are known to be associated with certain cancers, such as the anti‐NMDAR1 antibody and ovarian teratoma. Autoantibodies are hypothesized to cause direct neurotoxicity by downregulation of the target antigen. Although complement‐mediated damage also is possible, it is likely minimal and many other mechanisms potentially are involved.^30^, ^31^ The presence of anti‐NMDAR1 antibodies in serum of healthy humans, as well as dogs and cats, is well recognized and it has been suggested that neuronal cell surface antibodies are part of the physiological autoimmune repertoire.^32^ Moreover, the presence of CSF anti‐NMDAR1 antibodies has been associated with psychiatric signs in a mouse model without evidence of brain inflammation.^32^ The pathophysiological mechanisms by which these antibodies induce clinical disease remain to be uncovered and appear to be extremely complex. Importantly, these conditions are not always associated with MRI changes, as in 2 of the dogs we describe with anti‐NMDAR1 antibodies.^15^

Because of the difficulty in accessing CSF antibody testing in a timely fashion, and the importance of early intervention with immunomodulatory drugs and appropriate screening for neoplasia, clinical guidelines for recognition of AE in humans have been developed. These guidelines allow treatment to be initiated before detection of antibodies.^12^ However, a recent study highlighted the wide spectrum of presenting clinical signs, noting that clinical suspicion of encephalitis was present in only 16 of 50 patients that were ultimately diagnosed with AE. Of these 16 patients, an infectious cause was suspected in 9.^29^ Thus, AE was considered as a differential diagnosis at time of presentation and initial evaluation in only 7/50 (14%) of patients ultimately diagnosed with the condition.^29^ This observation highlights the importance of considering autoimmune mechanisms for CNS disorders currently believed to be idiopathic.

The expanding knowledge of neuronal surface antigen targets as a cause of AE in people, the recent publication on the presence of anti‐NMDAR antibodies in a polar bear,^21^ and the frequency with which MUE is diagnosed in dogs led us to investigate whether homologous autoantibodies against some of the more common human target antigens were present in dogs. We designed the study to screen CSF from dogs with a wide variety of neurological conditions, including conditions such as epilepsy and intervertebral disk extrusion as well as encephalitis to determine whether these autoantibodies were present in dogs with neurological disease, and with which diseases they were associated. Within this population of 32 dogs with neurologic disease and 1 healthy control, antibodies were not identified against AMPAR1/R2 or GABA_B_R1/R2 or CASPR2, LGI1, and DPPX in any dog. Three dogs had CSF that tested positive for antibodies against NMDAR1, the most common antigenic target associated with AE in people.^30^ The immunofluorescent staining in our positive canine samples was less intense than in the positive human control. This could mean that the antibody concentration was lower in the canine samples or that the canine CSF antibodies have a lower affinity for the NMDAR1 antigen used. It is possible but unlikely that these dogs possessed antibodies that cross‐reacted to bind antigens other than NMDAR1 because the most similar canine protein is NMDAR2B, which shares only 28% identity. Even if a dog possessed an anti‐NMDAR2B antibody that cross‐reacted to bind NMDAR1 in the assay employed in this study, both proteins are subunits of the heteromeric NMDA receptor complex and would not be considered a false‐positive for autoantibodies against the NMDA receptor. Nevertheless, it is recognized that there is a wide range of anti‐NMDAR antibodies with different target affinities in humans.^33^ Notably, dogs in our study that were diagnosed with brain neoplasia, idiopathic or unknown epilepsy, intervertebral disk extrusion and degenerative myelopathy did not have CSF antibodies to this target.

N‐methly d‐aspartate receptor encephalitis is well described in people and classically affects young women and children causing a rapid onset (defined as <3 months) of flu‐like clinical signs that progress to abnormal behavior or cognitive dysfunction, speech abnormalities, seizures, movement disorders (including dyskinesias and rigidity), decreased level of consciousness, and autonomic dysfunction. A fatal catatonic state can occur, but appropriate treatment with immunosuppression results in a favorable outcome.^15^, ^18^ A diagnosis of NMDARE can be made in patients with the rapid onset of 4 of the above 6 categories of neurological signs, and abnormal electroencephalography or CSF with pleocytosis or oligoclonal bands and reasonable exclusion of other disorders.^11^, ^12^, ^13^ It is notable that the syndrome is associated with teratomas in nearly 40% of patients and MRI findings are normal in 66% of patients.^15^, ^18^ Also of note is a genetic predisposition to the syndrome associated with a particular human leukocyte antigen 1 allele.^34^

The 3 dogs with CSF positive for anti‐NMDAR1 antibodies underwent extensive diagnostic evaluation. The first dog had a history and multifocal neurological signs typical of encephalitis as well as both multifocal MRI changes and CSF pleocytosis. The second dog had severe cerebellar signs, consistent with the atypical presentation reported in people more recently,^29^ as well as having CSF pleocytosis. In both dogs, infectious causes were excluded and their response to immunosuppression supports an autoimmune etiology although a causal relationship with the anti‐NMDAR antibodies cannot be established from these observations. The third case was more anomalous; as a 1‐year‐old spayed female French bulldog presenting with progressive, severe, multifocal CNS signs, the primary differential diagnosis at presentation was MUE. However, the dog's history was complicated by the presence of multiple abscessed teeth treated with broad‐spectrum antibiotics and recent airway surgery for brachycephalic airway syndrome. The normal appearance of the brain MRI and CSF analysis were puzzling given the severity of the neurological signs. In light of the signs of CNS dysfunction and the presence of resolving dental abscesses, treatment with antibiotics and prednisone at a dosage of 1 mg/kg/day was initiated. The dog's clinical signs responded to this treatment but relapsed as prednisone was tapered and then resolved with a longer course of prednisone. Although the dog's clinical diagnosis remains uncertain, the presence of CSF anti‐NMDAR1 antibodies suggests a possible autoimmune etiology, potentially triggered by the bacterial infection.

Our study had several limitations. Although high homology of all protein targets among species allowed for confidence in results, the commercially available kit used antigen targets designed for humans specifically and we did not confirm our findings by use of another assay. This project was developed to screen CSF banked from dogs with CNS dysfunction of a variety of causes for the presence of CSF antibodies against neuronal surface targets. Although the panel of antigenic targets was negative in dogs with epilepsy and degenerative conditions, the number of dogs with these conditions was limited and a much larger study of the prevalence of these antibodies in the CSF and serum of dogs with neurologic disease as well as the clinical relevance of these antibodies is warranted in light of our results. In addition, CSF and serum samples from a much larger cohort of neurologically normal dogs of a range of ages should be evaluated. Finally, the retrospective nature of our study means that dogs with CSF positive for antibodies were not followed prospectively and the presence of antibodies in serum was not evaluated.

Our results need confirmation by an additional assay or assays, but suggest that anti‐NMDAR1 antibodies can be found in the CSF of dogs with encephalitis. Although their pathologic role is unclear, these findings suggest that neuronal surface protein antibodies might be a cause of AE, and a much wider search for autoantibodies is warranted. Indeed, it is possible that dogs with idiopathic syndromes such as idiopathic cerebellitis (generalized tremor syndrome) or so‐called “fly biting” might have antibodies to an identifiable antigenic target in their CSF. Identification of these autoantibodies may allow better antemortem categorization of MUE as specific types of AE and improve our ability to provide a prognosis. A much larger prospective study evaluating a wide range of neuronal cell surface or synaptic antigenic targets is warranted.

## CONFLICT OF INTEREST DECLARATION

Authors declare no conflict of interest.

## OFF‐LABEL ANTIMICROBIAL DECLARATION

Authors declare no off‐label use of antimicrobials.

## INSTITUTIONAL ANIMAL CARE AND USE COMMITTEE (IACUC) OR OTHER APPROVAL DECLARATION

This study was conducted in accordance with guidelines and approval of the North Carolina State University IACUC (protocol number: 17‐053‐O).

## HUMAN ETHICS APPROVAL DECLARATION

Authors declare human ethics approval was not needed for this study.

## Supporting information


**Data S1:** Supporting Information, tables.

**Figure S1** Immunofluorescent assay results for the presence of CSF antibodies against AMPA receptors 1 and 2 (A), GABA receptors 1 and 2 (B), CASPR2 (C), LGI1 (D) and DPPX (E). Cellular immunostaining is uniformly absent in these samples although there is some artifactual staining of debris in D and E
