# Supplementary material for: Presence of cerebrospinal fluid antibodies associated with autoimmune encephalitis of humans in dogs with neurologic disease
Source: J Vet Intern Med. 2019 Sep 8;33(5):2175–82. doi: 10.1111/jvim.15616 (PMC6766506; doi:10.1111/jvim.15616)
Supplement: Supplementary file 1 — Data S1: Supporting Information, tables. [file JVIM-33-2175-s001.pdf]

ID	NMDAR	Age (y)	Breed	Sex	Presenting signs	CSF findings	MRI findings	Diagnosis	Treatment
1		13	Maltese	MC	Progressive tetra-ataxia, blind OU.	WBC: 197 cells/μL<br>RBC: 4 cells/μL<br>Protein: 30.3mg/dL<br>Moderate mononuclear pleocytosis	Symmetrical T2 hyperintesnwe non-contrast enhancing lesions, predominantly within the internal capsule and corona radiata.	MUE	Immunosuppression with prednisone and cytosine arabinoside.
3		6	French Bulldog	MC	Head tilt and tetraparesis.	WBC: 1 cells/μL<br>RBC: 0 cells/μL<br>Protein: 21.4mg/dL<br>Cytologically unremarkable	Multifocal poorly delineated T2 hyperintensities with mild contrast enhancement in the right frontal lobe, the thalamus, right mesencephalon and pons and diffuse meningeal enhancement	MUE	Immunosuppression with prednisone and cytosine arabinoside.
4	Y	6	Poodle (Mini)	FS	Torticollis, non-ambulatory tetraparetic	WBC: 1567 cells/μL<br>RBC: 1675 cells/μL<br>Protein: 674mg/dL<br>Mixed cell pleocytosis; increase in nucleated cells	Ill-defined bilateral T2 hyperintensity within thalamus, subthalamus, tegmentum and medulla.<br>There was no evidence of contrast enhancement.	MUE	Immunosuppression with prednisone and cytosine arabinoside.
5		5	Dachshund	FS	Head tilt, non-ambulatory tetraparesis.	*Lumbar*<br>WBC: 17 cells/μL<br>RBC: 50 cells/μL<br>Protein: 84mg/dL<br>Mild mononuclear pleocytosis	T2 hyperintense lesion of the right cerebellum and underlying brainstem. Strong contrast enhancement of the lesion with mild diffuse meningeal enhancement.	MUE	Immunosuppresion with prednisone and cyclosporin
6		4	Cavalier King Charles Spaniel	MC	Behavioral change, progressive tetraparesis and tetra-ataxia	*Lumbar*<br>WBC: 1702 cells/μL<br>RBC: 338 cells/μL<br>Protein: 188.9mg/dL<br>Marked lymphocytic pleocytosis	Chiari-like malformation with cerebellar herniation and cervical syringohydromyelia	Antemortem diagnosis: MUE, post mortem diagnosis: GME	Immunosuppresion with prednisone, arrested within 48 hours. Diagnosis of GME established at necropsy.

MC: male castrated; FS: female spayed
WBC: white blood cell; RBC: red blood cell

MUE: meningoencephalitis of unknown etiology
GME: granulomatous meningoencephalitis

7		3	Mixed Breed (Yorkie-poo)	MC	Seizures and behavioral changes.	WBC: 6 cells/µL<br>RBC: 33 cells/µL<br>Protein: 25.1mg/dL<br>Mild eosinophilic pleocytosis	Multifocal ill-defined T2 hyperintense parenchymal lesions within the cerebrum, with probably chronic necrosis and small regions of cavitation or subacute necrosis	MUE	Immunosuppression with prednisone and cytosine arabinoside.
10	Y	1	Standard Poodle	MC	Non-ambulatory tetraparesis with profound hypermetria and intention tremors	WBC: 43 cells/µL<br>RBC: 23 cells/µL<br>Protein: 52.6mg/dL<br>Lymphocytic pleocytosis	Unremarkable	MUE	Immunosuppression with prednisone.
11		6	Golden Retriever	MC	Head tilt, anxiety, pacing. Pre-existing reflex dyssynergia	WBC: 3 cells/µL<br>RBC: 0 cells/µL<br>Protein: 18mg/dL<br>One mitotic figure, otherwise cytologically unremarkable	Non-contrast enhancing, T2 hyperintense lesion extending from the left thalamus through the midbrain, brainstem and into the cranial cervical spinal cord without evidence of a mass effect	Suspected MUE	Immunosuppression with prednisone and azathioprine
12		4	Golden Retriever	MC	Lethargy, head tilt, circling and ataxia	WBC: 18 cells/µL<br>RBC: 3 cells/µL<br>Protein: 25.9 mg/dL<br>Mononuclear pleocytosis	Multifocal bilateral asymmetric T2 hyperintensity of the thalamus, midbrain and brainstem	MUE	Immunosuppression with prednisone and cytosine arabinoside.
15		5	Scottish Terrier	FS	Tetra-ataxia with left head tilt.	WBC: 1002 cells/µL<br>RBC: 3 cells/µL<br>Protein: 83.4mg/dL<br>Marked mixed pleocytosis	Plaque-like meningeal thickening and contrast enhancement along the left midbrain and brainstem	MUE	Immunosuppression with prednisone.
16		6	Pekapoo	FS	Blind, paraparesis and ataxia	*Lumbar*<br>WBC: 179 cells/µL<br>RBC: 17600 cells/µL<br>Protein: 232.3**mg/dL**<br>Lymphocytic pleocytosis	Marked meningeal enhancement throughout the thoracolumbar spinal cord, mild contrast enhancement of the intracranial meninges and of the hippocampus.	MUE	Immunosuppression with prednisone and cytosine arabinoside.
18		5	Japanese Chin	FS	Non-ambulatory paraparesis	WBC: 52 cells/µL<br>RBC: 445 cells/µL<br>Protein: 33.5**mg/dL**<br>Lymphocytic pleocytosis	Diffuse spinal cord swelling and T2 hyperintensity at T5-L3 with intramedullary and meningeal contrast enhancement	MUE	Immunosuppression with prednisone and cytosine arabinoside.

MC: male castrated; FS: female spayed
WBC: white blood cell; RBC: red blood cell

MUE: meningoencephalitis of unknown etiology
GME: granulomatous meningoencephalitis

19		8	Maltese	FS	Non-ambulatory tetraparesis, head tilt and torticollis to right.	WBC: 500<br>RBC: 360<br>Protein: 217.2<br>Marked mixed cell pleocytosis	Multifocal T2 hyperintense, contrast enhancing lesions intrparenchymal lesions affecting the cerebellum and cervical spinal cord. Moderate meningeal contrast enhancement.	MUE	Euthanasia, GME confirmed on histopathology
20		5	Maltese	MC	Spinal pain	*Lumbar*<br>WBC: 323 cells/μL<br>RBC: 5 cells/μL<br>Protein: 95.1mg/dL<br>Marked mononuclear pleocytosis	T2 hyperintensity of the brainstem and spinal cord with diffuse meningeal contrast enhancement	MUE	Immunosuppression with prednisone and cytosine arabinoside.
21		7	Maltese	MC	Blind, behavioral changes.	WBC: 7 cells/μL<br>RBC: 0 cells/μL<br>Protein: 28mg/dL<br>Mild mononuclear pleocytosis	Severe diffuse meningeal enhancement and multifocal meningeal thickening	MUE	Immunosuppression with prednisone.
22		3	Poodle-Toy	FS	Cluster seizures, non-ambulatory tetraparetic, behavioral changes.	WBC: 11 cells/μL<br>RBC: 55 cells/μL<br>Protein: 30mg/dL<br>Mild mononuclear pleocytosis	No MRI	GME	Euthanized, diagnosis established on histopathology
23		7	Goldendoodle	FS	Cerebellar ataxia with intention tremors and vertical nystagmus.	WBC: 3 cells/μL<br>RBC: 30 cells/μL<br>Protein: 22mg/dL<br>Cytologically unremarkable.	Multifocal poorly delineated areas of T2 hyperintensity and contrast enhancement in the cerebellum and left brainstem	MUE	Immunosuppression with prednisone and cytosine arabinoside.
2		9	Labrador Retriever	FS	Cerebellar ataxia and generalized intention tremors.	WBC: 4 cells/μL<br>RBC: 20 cells/μL<br>Protein: 13.5mg/dL<br>Cytologically unremarkable	Unremarkable	Cerebellitis	Methocarbamol; Immunosuppression with prednisone

MC: male castrated; FS: female spayed
WBC: white blood cell; RBC: red blood cell

MUE: meningoencephalitis of unknown etiology
GME: granulomatous meningoencephalitis

**14**		8 months	Bernese Mountain Dog	FS	Spinal pain	WBC: 74 cells/μL<br>RBC: 8 cells/μL<br>Protein: 36.4 mg/dL<br>Marked neutrophilic pleocytosis	Meningeal contrast enhancement	SRMA	Immunosuppression with prednisone.
**17**	Y	1	French Bulldog	FS	Circling to right, right head tilt, behavioral changes, right hemiparesis	WBC: 2 cells/μL<br>RBC: 48 cells/μL<br>Protein: 12.2mg/dL<br>Cytologically unremarkable	**Normal CNS structures on MRI. Tooth root abscesses in left maxilla extending into nasal cavity via a fistula.**	No neurologic diagnosis made. Tooth root abscess	Prednisone 1mg/kg/day and antibiotics
**8**		13	Dachshund	MC	Seizures	WBC: 10244 cells/μL<br>RBC: 1450 cells/μL<br>Protein: 146.8mg/dL<br>Marked neutrophilic pleocytosis	Peripherally contrast enhancing, presumed extra-axial mass in the left frontal lobe and mild diffuse meningeal enhancement	Neutrophilic meningoencephalitis and Brain tumor, suspect meningioma	Anti-inflammatory doses of prednisone, treatment of possible infectious meningitis with trimethoprim sulfa and doxycycline. Levetiracetam to treat the seizures.
**29**		10	Golden R	MC	Seizures.	WBC: 0 cells/μL<br>RBC: 0 cells/μL<br>Protein: 23.6mg/dL<br>Interpretation: Increased percentage of neutrophils with cell count and total protein within reference limits	Contrast enhancing, T2 hyperintense mass affecting the right olfactory bulb, with a prominent dural tail along the falx cerebri and associated with 2 cystic structures.	Suspected brain tumor: cystic meningioma	Prednisone and levetiracetam

MC: male castrated; FS: female spayed
WBC: white blood cell; RBC: red blood cell

MUE: meningoencephalitis of unknown etiology
GME: granulomatous meningoencephalitis

30		8	Mixed Bre	FS	Abnormal mentation	WBC: 30 cells/μL<br>RBC: 1380 cells/μL<br>Protein: 27mg/dL<br>Neutrophilic pleocytosis	Severe swelling and T2 hyperintensity of the right cerebral hemisphere with possible subependymal infiltration. Lateral ependymal contrast enhancement and equivocal right sided meningeal contrast enhancement. Secondary transtentorial herniation.	Anaplastic oligodendro glioma	Euthanized, diagnosis established on histopathology
9		11	Boston Terrier	MC	Seizures and behavioral changes, tetraparesis and torticollis.	WBC: 7 cells/μL<br>RBC: 265 cells/μL<br>Protein: 23mg/dL<br>Possible mild mononuclear cell pleocytosis	Multifocal T2 hyperintense non-contrast enhancing lesions of the thalamus and midbrain.	Brain tumor: Gliomatosis cerebri	Immunosuppression with prednisone and cytosine arabinoside, no response. Euthanized, diagnosis established at necropsy.
24		6	America n Stafford shire Terrier	MC	Cluster seizures.	WBC: 20 cells/μL<br>RBC: 45 cels/μL<br>Protein: 103.7mg/dL<br>Mild neutrophilic pleocytosis	Bilateral extensive and expansile intra-axial T2 hyperintense lesions affecting predominantly white matter within the piriform lobes, hippocampus, internal capsule, and frontal lobes. Patchy contrast enhancement.	DDx MUE; gliomatosis cerebri; round cell neoplasia	Euthanized, no necropsy
25		4	Golden Retrieve r	MC	Seizures.	WBC: 1 cells/μL<br>RBC: 0 cells/μL<br>Protein: 23.1mg/dL<br>Cytologically unremarkable CSF	Mild widening of sulci with cortical thinning.	Idiopathic Epilepsy	Continued anti-epileptic drugs
26		8	Schnauz er	FS	Seizures.	WBC: 1<br>RBC: 0<br>Protein: 19.8<br>Cytologically unremarkable CSF	Unremarkable	Idiopathic Epilepsy	Continued anti-epileptic drugs
27		6	Mixed Bre	MC	Seizures	WBC: 1 cells/μL<br>RBC: 0 cells/μL<br>Protein: 10mg/dL<br>Cytologically unremarkable	Unremarkable	Idiopathic Epilepsy	Continued anti-epileptic drugs

MC: male castrated; FS: female spayed
WBC: white blood cell; RBC: red blood cell

MUE: meningoencephalitis of unknown etiology
GME: granulomatous meningoencephalitis

13		10	XL Mixed Breed	MC	Generalized seizures	WBC: 1 cells/μL<br>RBC: 3 cell/μL<br>Protein: 24.5mg/dL<br>Interpretation: Cytologically unremarkable	Unremarkable	Unknown Epilepsy	Levetiracetam
28		7	Beagle	MC	Ambulatory paraparesis with spinal pain	WBC: 1 cells/μL<br>RBC: 3 cells/μL<br>Protein: 20.7mg/dL<br>Cytologically unremarkable	Right lateralized chronic intervertebral disc herniation T12-13 with severe extradural spinal cord compression and focal spinal cord gliosis	Chronic IVDE	Surgical decompression.
31		10	Labrador	MC	Progressive paraparesis	*Lumbar*<br>WBC: 2 cells/μL<br>RBC: 123 cells/μL<br>Protein: 89.4mg/dL<br>Albuminocytologic dissociation	Spinal MRI within normal limits.	Degenerativ e myelopathy	
32		4	Labradoo	MC	Paraplegia	*Lumbar*<br>WBC: 71 cells/μL<br>RBC: 1167 cells/μL<br>Protein: 417.4mg/dL<br>Mixed cell pleocytosis	Dorsal, right and left dorsolateral extradural material T10 to L2 and ventrally located herniated disc material at T12-13 causing multifocal mild spinal cord compression	IVDE	Surgical decompression.

MC: male castrated; FS: female spayed
WBC: white blood cell; RBC: red blood cell

MUE: meningoencephalitis of unknown etiology
GME: granulomatous meningoencephalitis
