# Supplementary figures and images for: Presence of cerebrospinal fluid antibodies associated with autoimmune encephalitis of humans in dogs with neurologic disease
Source: J Vet Intern Med. 2019 Sep 8;33(5):2175–82. doi: 10.1111/jvim.15616 (PMC6766506; doi:10.1111/jvim.15616)

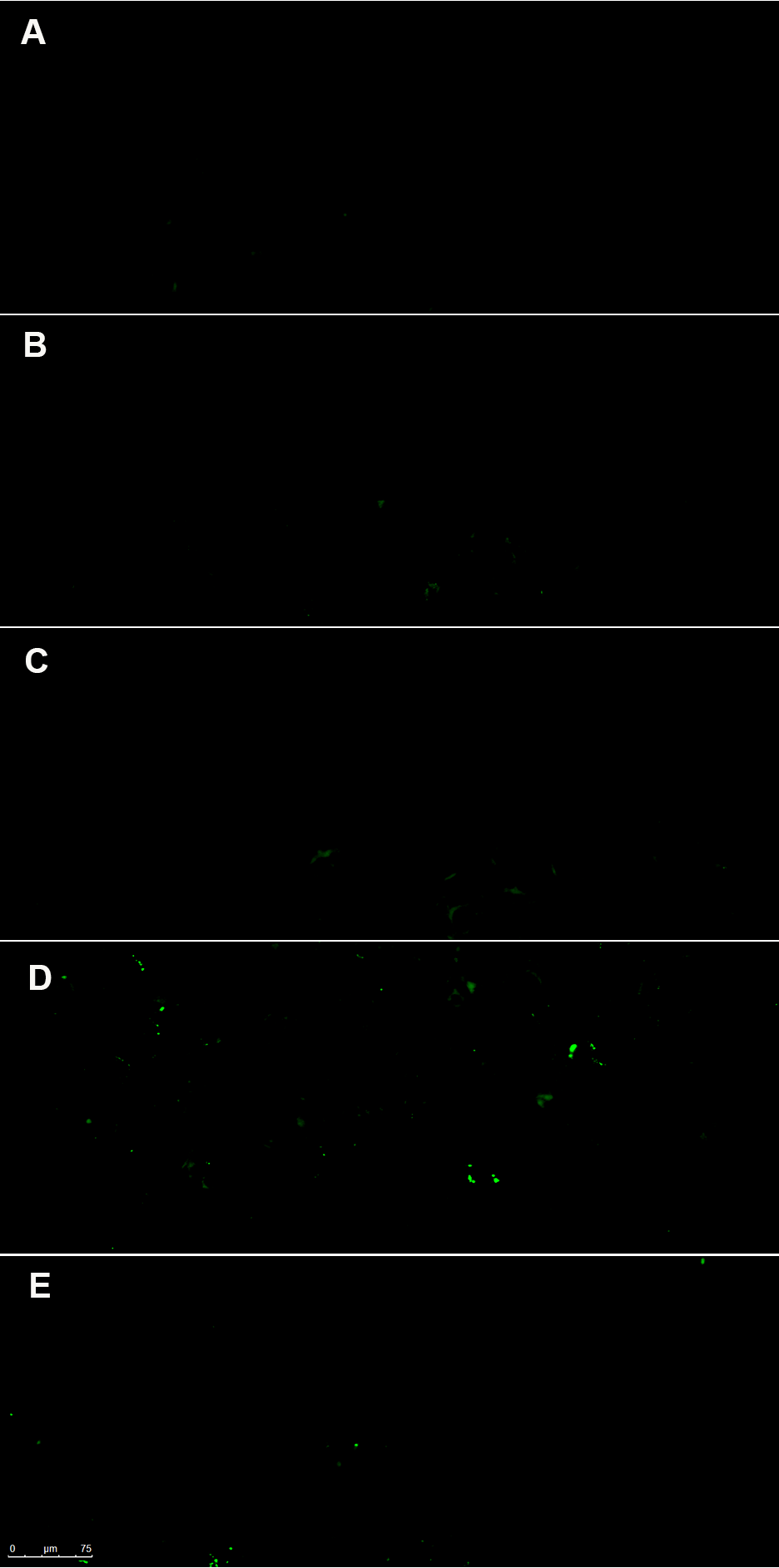

Supplement: Supplementary file 2 — Figure S1 Immunofluorescent assay results for the presence of CSF antibodies against AMPA receptors 1 and 2 (A), GABA receptors 1 and 2 (B), CASPR2 (C), LGI1 (D) and DPPX (E). Cellular immunostaining is uniformly absent in these samples although there is some artifactual staining of debris in D and E [file JVIM-33-2175-s002.tif]
